# Pressure Ulcer Risk Evaluation in Critical Patients: Clinical and Social Characteristics

**DOI:** 10.2174/1874434601711010091

**Published:** 2017-07-28

**Authors:** Mônica Suêla de Azevedo Macena, Rayanne Suely da Costa Silva, Maria Isabel Da Conceição Dias Fernandes, Ana Beatriz de Almeida Medeiros, Kadyjina Daiane Batista Lúcio, Ana Luisa Brandão de Carvalho Lira

**Affiliations:** Nursing Department, Federal University of Rio Grande of Norte, Natal, Rio Grande of Norte, Brazil

**Keywords:** Nursing, Inpatients, Intensive care units, Skin, Pressure ulcer, Risk factors

## Abstract

**Background:**

Pressure ulcers increase hospital stays and treatment costs due to their complications. Therefore, recognizing factors that contribute to pressure ulcer risk are important to patient safety.

**Objective:**

To evaluate the association between the scores of the Waterlow, Braden, and Norton scales and clinical and social characteristics in critically ill patients.

**Method:**

A cross-sectional study of 78 patients in an adult intensive care unit of a university hospital in Northeastern Brazil was conducted from July to December 2015. Data included social and clinical information and the risk factors of the Braden, Norton and Waterlow scales. Data were analysed by the descriptive and inferential statistics.

**Results:**

Most of the participants were female, adults and elderly people with brown skin colour, low education levels and insufficient income. Most of them showed a high risk for developing pressure ulcers using the three evaluated scales. Age, smoking status, diabetes and hypertension were associated with scores on the Waterlow, Braden and Norton scales.

**Conclusion:**

Age, use of the tobacco, diabetes and hypertension were associated with the risk of pressure ulcers in ICU patients.

## 1 INTRODUCTION

The intensive care unit (ICU) is intended for clinically unstable patients who need intermittent care and technology to evaluate and control their vital functions [1]. In this unit, pressure ulcers have an incidence of 23.1% [2]. Several risk factors contribute to skin damage in these critically ill patients, including nutritional deficits, decreased tissue perfusion, long-term use of a mechanical ventilator, the presence of moisture and circulatory changes [3].

Pressure ulcers (PUs) are injuries that originate in the epithelial tissue and may reach lower layers, such as vessels, muscles and bones [4, 5]. PUs develop with tissue pressure over a bony prominence that has an intensive capability of collapsing the capillary of that region, and in association with friction and shear, abrasion may occur, facilitating the onset of injuries [4, 5].

PUs are complications that can worsen the clinical conditions of severely ill patients. Ulcers lead to infections and to an increase in microbiological resistance. Mortality is related to the worsening of clinical conditions. PUs increase the period of hospitalization and the costs of treatment due to the resulting complications [6].

After 15 days of hospitalization in an ICU, all patients have some risk for developing a PU, especially bedbound elderly patients [7]. The expenditures on treatment materials exceed $300.00 per day, totalling $111.416.07 per year [8]. The reduction of the incidence of PUs can decrease hospital costs and optimize the care provided by the nursing staff [9].

Therefore, preventive actions are necessary to be taken in the ICU, which can help nurses avoid the occurrence of this adverse event [10]. Health professionals, especially the nursing staff, focus on improving human health conditions. Consequently, avoidance of adverse events related to the care provided should be ensured [11].

The following are among the main interventions by nurses to prevent pressure ulcers: changing position every 2 hours, comfort massage, the use of pyramidal mattress, proper nutrition, and physical examination. However, knowledge about risk factors is necessary to recognize and direct nursing actions to prevent PUs [12]

The risk of developing a PU can be evaluated from measurement scales of pressure ulcer risk factors [13]. Currently, three scales are highlighted for this purpose, the Waterlow, Braden, and Norton scales [14]. These scales may predict the potential for each patient to develop the skin trauma.

Therefore, recognition of risk factors for critically ill patients can help reduce the risk of PUs [15]. A decrease in the occurrence of these lesions optimizes nursing care delivery and improves the quality of life of the hospitalized patient. For this, nurses should conduct periodic evaluations to identify the risk of PUs and improve their knowledge about the use of measurement scales for PUs [13]. Furthermore, the nurse must evaluate the patient's clinical and social characteristics to understand the patient´s clinical and social context in the risk of developing a PU.

The assumption of this study is that individuals with hypertension, diabetes, advanced age and smokers have the highest risk of developing pressure ulcers. Therefore, the objective of this study was to evaluate the association between the Waterlow, Braden, and Norton scale scores and the clinical and social characteristics in critically ill patients.

## 2 MATERIALS AND METHOD

### 2.1 Design

A cross-sectional study was conducted on patients admitted in the adult ICU of a university hospital in Northeast Brazil. The setting involved a general ICU with 19 hospitals beds having patients who suffer from injuries, such as cardiovascular illness, respiratory disease, cancer, renal dysfunction and sepsis. Selection of this ICU was supported by the quality of the unit as a reference for the care of critically ill patients.

### 2.2 Sample

The sample was determined from the application of a formula developed for studies with finite populations: n = Z∞^2^ * P * Q * N / E^2^ * (N-1) + Z∞^2^ * P * Q [16]. The parameters used in the calculation of the sample were as follows: n being the sample size, Z being the confidence level of the study (Z∞ = 1.96), E as the sample error (E = 10%), N as the population size (N = 883), P as the prevalence of pressure ulcers in patients in the intensive care unit (P = 57.89%), and Q being the complement of the prevalence (100 - P). Seventy-eight individuals were identified from the application of the formula. The sample calculation was performed prior to data collection.

The sample met the following inclusion criteria: patients with clinical or surgical treatment in the ICU older than 18 years of age. Patients admitted to the ICU without a pressure ulcer were excluded from this study. The sample was selected based on a non-probability sampling method with selection for convenience.

### 2.3 Instrument and Data Collection

The data collection instrument contained socioeconomic (sex, race, education, family income, employment status and age) and clinical information (length of hospital stay, reason for hospitalization, diabetes, hypertension, alcoholism and smoking) as well as the risk assessment scales for PU and their respective variables, including the Braden (sensory perception, moisture, activity, mobility, nutrition and friction/shear); Norton (physical condition, mental state, activity, mobility and incontinence); and Waterlow (body mass index, skin type, sex, age, continence, mobility, malnutrition of the cellular tissue, neurological deficiency, major surgery/trauma, appetite and medication) scales [13]. Age was a possible confounding variable because older individuals are at an increased risk of developing pressure ulcers.

The Braden scale is comprised of six pressure ulcer risk factors. Each factor is classified by scores. The total final sum of the scores provides the risk classification for the evaluated patients, as follows: very high risk: 6-9 points, high risk: 10-12 points, medium risk: 13-14 points and low risk: 15-18 points [13, 14]. The Norton scale consists of five risk factors that have been classified into scores that provide the final classification of risk for PU, as follows: high risk: less than or equal to 12 and low risk: greater than 12 points [13, 14]. The Waterlow scale is divided into 11 risk factors that allow for assessment of the risk of PU. To this end, it provides the following classifications: at risk: above 10; high risk: above 15; and very high risk: over 20 [13, 17].

Final classifications of the three scales were dichotomized into high and low risk for the development of PU. This division was adopted to standardize the results of the three scales. Therefore, for the Braden scale, patients with scores ≥ 15 were classified as low risk and those with scores ≤ 14 were classified as high risk. For the Waterlow scale, the classification of the scores was done as follows: patients with scores > 10 were classified as having high risk and those with scores ≤ 10 were at low risk. The Norton scale remained unchanged.

Data collection was performed by two nursing students and one resident nurse. During the collection, each patient was evaluated by a single collector. For standardization, the collection was performed with previous training of the data collectors. The discussed points were intensive care, critically ill patients, pressure ulcers and their risk factors, and all the items in the collection instrument were explained. After training, data collection was performed between the months of July to December 2015.

### 2.4 Data Analysis

Data were organized in Microsoft Excel and analysed in SPSS version 19.0 for Windows Statistic. The relative and absolute frequencies were stipulated for nominal variables. For the numerical variables, the mean and standard deviation were determined. Inferential statistics were also used, including the chi-square, Fisher’s exact and Mann-Whitney U tests. A p-value of <0.05 was adopted. Scales used to verify the risk of PU were dichotomized as high and low risk to allow for application of the aforementioned tests.

### 2.5 Ethical Consideration

The research followed the ethical and legal aspects for its development, as required by the Brazilian Resolution number 466/12 of the Brazilian National Health Council that oversees research involving human beings. This study was approved by the ethics committee of the university hospital. The approval number for this research is 848.997/2014. Patients were informed of the purpose of the research, method and possible risks, and they were asked to sign an informed consent form.

## 3 RESULTS

Regarding the patients participating in the study, 55.1% were female, of whom 50% had brown skin. Of the participants, 44.9% reported having incomplete primary education, and 19.2% were illiterate. Most of the family income (71.8%) was from one to three minimum wages, and 56.4% were retired. In relation to age, there was an average of age of 58.3 (±17.2) years.

The hospitalization time was 1 to 44 days with a mean of 17.2. Regarding the reasons for hospitalization in the ICU, there were 41 (48.2%) surgical cases; 14 (16.5%) cardiac cases; 13 (15.3%) pulmonary disorders and 17 (20%) with hepatic, renal and neurological problems. Hypertension was present in 56.4% and diabetes in 29.5% of cases. A minority reported smoking (15.4%) and drinking (6.4%).

Most patients had a high risk for developing of PU according to the Braden (74.4%), Norton (70.5%) and Waterlow (62.8%) scales, as shown in Table **1**.

In relation to scales’ scores and social and clinical data of the patients admitted to the ICU, there was a significant association between the age, hypertension, diabetes, and smoking status and the Waterlow scale. In addition, smoking was significantly associated with the Braden and Norton scales, as shown in Table **2**.

## 4 DISCUSSION

Sex-related data were similar to another study with an incidence of 52.6% of female patients [18]. Women are at an increased risk for developing PU, which is in part due to their higher level of adipose tissue, placing increased pressure on the tissues and resulting in cell hypoxia [19].

A low educational level and income were observed in this research study, which may be related to the evaluated ICU. The ICU is a public institution that treats patients with low purchasing power and few years of study. Low socioeconomic status is evidenced in the literature as a characteristic of patients with PU risk [20]. This result indicates the necessity for nurses to develop health education to prevent PU in this population.

Regarding race, brown individuals comprised the majority in this study, although race cannot be considered alone as a risk factor for PU [18]. Regarding the age group, the majority of the patients were approximately 60 years old, and there was a predominance of surgical patients, which agrees with the literature data [7, 21].

In this study, most patients had a high risk for developing PU. Another study showed that 67% of ICU patients have a high risk for developing ulcers, supporting the data found in the present study [22]. Among the scales used in this study, the Braden scale indicated a higher risk for developing PU in the studied patients. Risk assessment scales are used to minimize the incidence of PU from the early identification of the risk. Among the existing scales, Braden is the most cited in the literature [13] and is recommended by the Ministry of Health protocol for pressure ulcer prevention [23].

On the other hand, another study [14] demonstrated that the Waterlow scale has high sensitivity (50.6%) and specificity (60.1%) compared to the Braden and Norton scales. Waterlow is the only scale among the three that presents the clinical evaluation of the skin as a risk factor for developing a lesion. In addition, it is the only one of the three that assesses age as a risk factor for developing a lesion. The authors [24] argue that the relationship between increased risk and increased age is due to changes in the skin and subcutaneous tissue characteristics of the elderly that become fragile and susceptible to pressure, friction and shear forces.

Finally, the Norton scale, compared to the others, is less effective in identifying the risk of PU because it does not have specificity in the scores; therefore, it is more subjective [13]. However, this report from a previous study does not agree with the findings of the present study.

Regarding the associations between social and clinical characteristics and the scales surveyed in the sample of patients, there was an association between the age, hypertension, diabetes, smoking status and the Waterlow scale. In this sense, the average age of the individuals was 58.3 years old, which confirmed the prevalence of adults and elderly people in the ICU. Older people have decreased adipose tissue and collagen fibres with a reduced blood supply from the decreased capillaries in the skin, which interferes with adequate blood perfusion [25]. Evidence confirms a possible relationship between age and a high risk of PU.

Additionally, non-communicable chronic diseases are often identified in the elderly, which clinically contributes to the emergence of PUs [26]. In this respect, together with the age, high blood pressure was another common clinical indicator in these subjects. Most patients with hypertension had a high risk of pressure ulcers according to the Waterlow scale, and there was a statistically significant association.

Hypertension causes stiffness in the blood vessels, decreasing the amount of blood in the tissue. This process increases with vascular ageing, favouring the development of PU. Damage to a target organ, such as the heart, brain, and kidneys, also contributes to the pathophysiology of pressure ulcers [26]. Nurses should consider this finding and provide measures to prevent PUs for patients with these characteristics in the ICU.

Diabetes, another indicator associated with the Waterlow scale, is prevalent in 6.2% of Brazilians and affects 387 million people worldwide [27]. Diabetes is responsible for causing metabolic, macro and microvascular disorders because of chronic hyperglycaemia. Therefore, there is a formation of advanced glycation end products (AGEs) and deposits of low density lipoprotein (LDL) according to elevated blood glucose, which causes tissue and cellular damage and deposition of atheromatous plaques in blood vessels, decreasing the amount of blood to the tissues, thus favouring the emergence of PU [28].

Smokers also had higher scale scores in this study. Although the number of smokers in this study was low, the number of smokers in Brazil has reached up to 10% of the total population [29]. Prior studies have revealed the relationships between smoking and an increased risk for the development of PU. As addiction caused by nicotine leads to cigarette use for many years, and the long-term consequences of smoking include cardiorespiratory problems. Cardiorespiratory symptoms lead to poor tissue oxygenation and a decrease in blood perfusion, allowing for the emergence of PU in oxygen-poor areas [30].

Thus, a plan of nursing care should consider the actual clinical and social needs of critically ill patients hospitalized in the ICU when preventing PU, acting as the main risk factor. Nursing care can improve patient safety care and reduce hospitalization costs.

### 4.1 Limitations

The present study has some limitations. First, a convenience sample was used. Another limitation is the use of scales (Waterlow and Braden) with results dichotomized into high and low risk, which may have caused bias in the research. Finally, this study included a cross-sectional design, and it was unable to examine the causal effect of the relationship between the variables. Therefore, further studies are recommended to more precisely confirm those relationships.

## CONCLUSION

Age, tobacco use, diabetes and hypertension are associated with the risk of developing pressure ulcers in ICU patients. This finding confirms the study assumptions. Most patients had a high risk for developing a PU. Among the scales, Braden indicated a higher risk for the development of PUs in the studied patients.

The practical implications of this study are that nurses should use pressure ulcer risk assessment scales in ICU patients as well as pay attention and implement preventive measures for patients admitted to the ICU considered as risk groups, such as elderly and patients with diabetes, hypertension and a smoking history.

## Figures and Tables

**Table 1:** Distribution of the Braden, Norton and Waterlow scale scores for the patients admitted to the ICU (n=78).

**Scale**	**Low risk**		**High risk**	
	**N**	**(%)**	**n**	**(%)**
Braden	20	25.6	58	74.4
Norton	23	29.5	55	70.5
Waterlow	29	37.2	49	62.8

**Table 2:** Distribution of the Norton, Braden and Waterlow scale scores and clinical and social characteristics of patients admitted to the ICU (n=78).

**Variable**	**Gender**		**Marital Status**		**Age**	**Religion**		**Hypertension**		**Diabetes**		**Alcohol consumption**			**Smoking**		
**Norton Scale**	Female	Male	With a partner	Without a partner	-	Practising	Not Practising	Yes	No	Yes	No	Yes		No	Yes		No
High Risk	34	21	37	18	-	49	06	32	23	16	39	03		52	05		50
Low Risk	09	14	15	08	-	20	03	12	11	07	16	02		21	07		16
**p-value**	0.066^1^		0.861^1^		0.149^3^	0.530^2^		0.626^1^		0.906^1^		0.464^2^			**0.024^2^**		
**Braden scale**																	
High Risk	33	25	37	21	-	51	07	35	23	19	39	04	54		06	52	
Low Risk	10	10	15	05	-	18	02	09	11	04	16	01	19		06	14	
**p-value**	0.593^1^		0.359^1^		0.212^3^	0.582^2^		0.233^1^		0.281^1^		0.619^2^			**0.046^2^**		
**Waterlow scale**																	
High Risk	26	23	31	18	-	42	07	33	16	20	29	03	46		04	45	
Low Risk	17	12	21	08	-	27	02	11	18	03	26	02	27		08	21	
**p-value**	0.633^1^		0.407^1^		**0.004^3^**	0.274^2^		**0.011^1^**		**0.004^1^**		0.619^2^			**0.026^2^**		

**Legend: ^1^**Chi-square test; ^2^Fisher’s Exact Test; and ^3^Mann-Whitney U test.
